# Supplementary material for: Aedes aegypti Beta-1,3-Glucan-Binding Protein Inhibits Dengue and ZIKA Virus Replication
Source: Biomedicines. 2024 Jan 1;12(1):88. doi: 10.3390/biomedicines12010088 (PMC10812959; doi:10.3390/biomedicines12010088)
Supplement: Supplementary file 1 [file biomedicines-12-00088-s001.zip › biomedicines-2742288-supplementary.pdf]

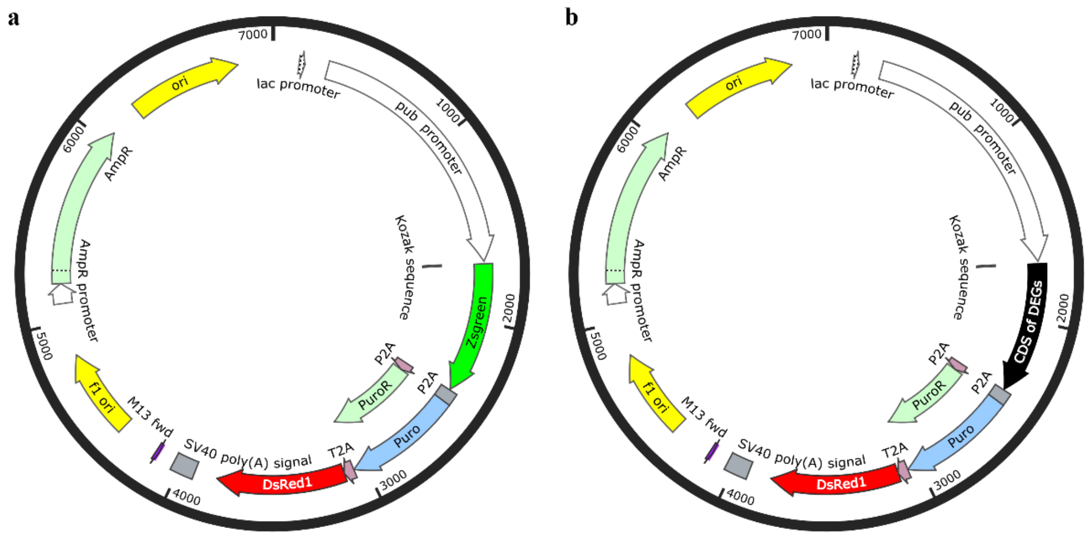

Figure S1. Constructed control plasmid vector (EGFP) (a) and experimental plasmid vector (b). Pub promoter is the polyubiquitin promoter sequence of Aedes mosquito, which has significant effects in enhancing the long-term and stability of gene expression. Zsgreen means Enhanced Green Fluorescent Protein (EGFP). DsRed means Red Fluorescent Protein. CDS of DEGs represents the protein coding sequences of *GNBPB6*.
